# Implementation of Pectointercostal Fascial Plane and Rectus Sheath Block as a Tool in Weaning From Mechanical Ventilation in Gripping Poststernotomy Pain: A Case Report

**DOI:** 10.1155/cria/4642503

**Published:** 2026-01-28

**Authors:** Mohammad Zishan Uddin, Maliha Karim, Md. Abdul Karim, Niaz Ahmed, Saiful Islam Khan

**Affiliations:** ^1^ Department of Cardiothoracic-Vascular Anesthesia & ICU, Evercare Hospital, Dhaka, Bangladesh; ^2^ Department of Anesthesia, Analgesia & ICU, Sir Salimullah Medical College Hospital, Dhaka, Bangladesh; ^3^ Department of Cardiothoracic Anesthesia & ICU, Labaid Cardiac Hospital, Dhaka, Bangladesh

**Keywords:** mechanical ventilation, pectointercostal fascial block, poststernotomy pain, rectus sheath block, weaning failure

## Abstract

Successful weaning from mechanical ventilation depends on many factors. Optimizing all factors creates the pathway of liberation from ventilation. Among them, comprehensive pain management is one of the most critical factors, especially for cardiac surgical patients, who require median sternotomy. This case report is about a case of a 55‐year‐old patient with diabetes mellitus, hypertension, and pneumonia, in whom weaning failed repeatedly after a coronary artery bypass graft operation and was successfully extubated after ultrasound‐guided implementation of a combined bilateral pectointercostal fascial plane block and rectus sheath block by reducing pain and improving ventilation, which was evidenced by reduced visual analog scale score and improvement of tidal volume. Integration of regional anesthesia not only provides adequate analgesia but also reduces opioid requirements and facilitates weaning, particularly for postcardiac surgical patients.

## 1. Introduction

Dire postoperative pain is one of the significant medical problems [1]. In most cardiac surgeries, a median sternotomy is the preferred surgical incision. If sternotomy pain is not adequately managed, it can lead to other medical problems, such as pneumonia, hypercoagulability, delirium, cardiovascular complications, wound infections, and so many others [2]. Significant pulmonary impairments may occur following median sternotomy and may become chronic pain [3, 4]. All these increase the respiratory load, making weaning from mechanical ventilation more difficult [5]. Systemic usage of opioids has been the mainstay of pain management for cardiac surgical patients for a long time. Opioid usage comes with side effects such as respiratory depression, sedation, nausea, vomiting, and ileus [1]. All these causes prolonged mechanical ventilation and delayed weaning from the ventilator. In the case of a postcardiac surgical patient, neuraxial anesthesia is debatable due to concerns regarding anticoagulant and antiplatelet therapy [6]. The implementation of regional anesthesia can alleviate all these problems. For median sternotomy pain management, ultrasound‐guided pectointercostal fascial plane block (PIFB) is a relatively promising one, which can be supplemented with rectus sheath block (RSB) for a greater outcome. The patient’s attendees in this case report provided written consent to both the intervention and publication. The patient was also informed and gave permission. This case highlights the potential role of regional anesthesia as one of the rescue strategies in patients with persistent weaning failure due to severe poststernotomy pain.

## 2. Case Presentation

**FIGURE 1:**
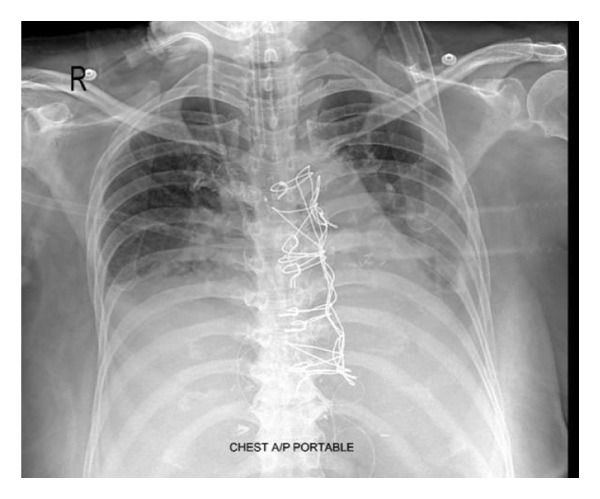
Chest X‐ray AP view showing sternal wiring with Robicsek technique.

**FIGURE 2:**
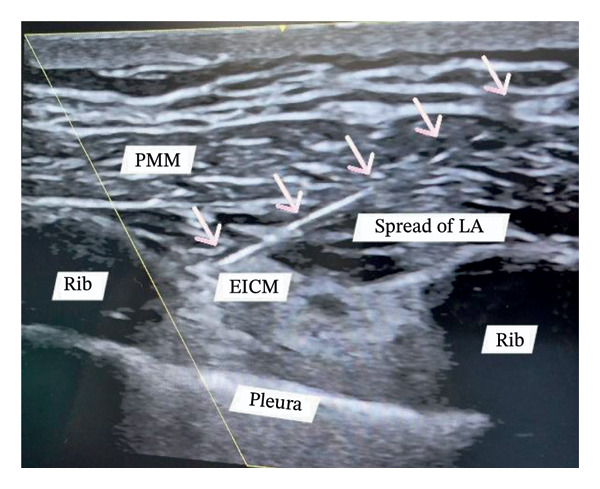
Pectointercostal fascial plane block (PIFB). Long axis technique. The needle tip is positioned between the pectoralis major muscle (PMM) and the external intercostal muscle (EICM). White arrows indicate the pathway of the entire needle. Both muscle layers are separated by the spread of local anesthesia (LA). The oblique yellow line indicates the activation of the needle recognition software for better visualization of the needle.

A 55‐year‐old female, diabetic, hypertensive patient underwent coronary artery bypass grafting (CABG) for triple vessel disease via median sternotomy in a hospital in Dhaka city, Bangladesh. The surgeon used the Robicsek technique for sternal reinforcement (Figure 1), and three chest drainage tubes were placed through the epigastric area. The patient was extubated the next day. However, the patient was reintubated again 24 h later. For two consecutive weeks, a weaning trial was given. However, extubating was not possible. On the 15th day, the patient was shifted to the ICU of Evercare Hospital, Dhaka, Bangladesh. The patient was previously diagnosed with pneumonia and treated with appropriate antibiotics. The patient was put on synchronized intermittent positive pressure ventilation (SIMV). Oxygen requirement was minimal. The patient was fully conscious. Glasgow Coma Scale was E4VTM6. Pain was excruciating, which mainly felt in the left parasternal and epigastric areas. A visual analog scale (VAS) was used for the pain assessment, with a rating of 10/10. A high dose of opioids was needed to minimize pain. Morphine infusion was used both for sedation and analgesia. All vitals were within normal range. The patient was hemodynamically stable, euglycemic, with regular arterial blood gas (ABG) values, adequate urine output, normal complete blood count, and liver function test values. The next day’s chest X‐ray showed improved consolidation; all hemodynamic parameters were normal with minimal oxygen support. Morphine infusion was stopped. So, a weaning trial was given. However, the trial failed due to restricted chest movement for severe pain, while the patient was on pressure support ventilation despite high pressure support. Differential considerations for weaning failure included respiratory muscle weakness and sedation effects. The patient was back on SIMV mode, and morphine infusion was initiated again. To reduce opioid dependency, for optimum pain management purposes, a combination of PIFB and RSB was planned for the next day. On the scheduled day, under strict aseptic conditions, we placed bilateral PIFB and subxiphoid RSB using a Venue Go ultrasound machine. Each block site was locally anesthetized with 1–2 mL of 2% lidocaine using a hypodermic needle. A high‐frequency linear probe was placed just 2 cm lateral to the sternum in a longitudinal manner over ribs three and five. After identifying the costal cartilage, pectoralis major muscle, and pleura, a 21‐gauge, 100 mm atraumatic needle was used for the block, performed using the in‐line technique in a caudal‐to‐cephalic direction. Needle recognition software was activated prior to needle insertion for optimal visualization of the needle. The needle was placed between the pectoralis major and the external intercostal muscle. A test bolus of normal saline was used to identify the needle tip. After negative aspiration, the drug was injected into the desired space. Real‐time spread of the drug in the fascial plane was observed in the ultrasound machine (Figure 2). The same procedure was similarly applied on the opposite side.

**FIGURE 3:**
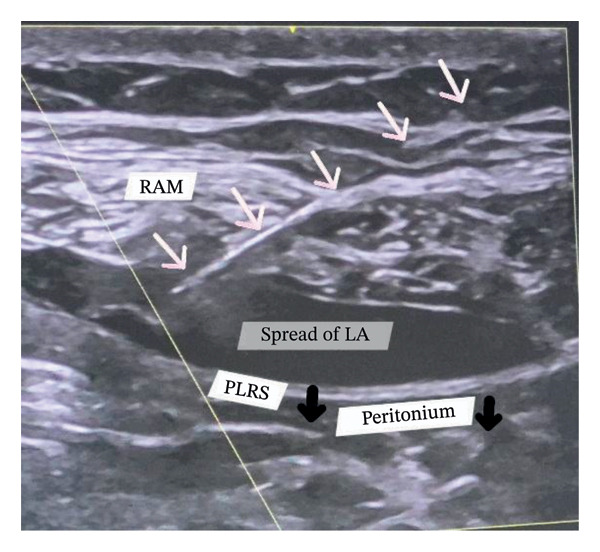
Rectus sheath block (RSB). Long axis technique. The needle tip is placed between the rectus abdominis muscle (RAM) and the posterior layer of the rectus sheath (PLRS). Both are separated by the spread of local anesthesia (LA), causing downward traction of the PLRS and the peritoneum (indicated by the black arrows). White arrows indicate the pathway of the entire needle. The oblique yellow line indicates the activation of the needle recognition software for better needle visualization.

For RSB, a high‐frequency linear probe was placed transversely in the epigastric area, just 3 cm below the xiphoid process. The rectus abdominis muscle, the rectus sheath with both layers, and the peritoneum were identified. In the long‐axis technique, the needle tip was positioned in the targeted space. Needle tip position was confirmed by hydrolocation. After negative aspiration, the drug was injected, and its real‐time spread was monitored by separating the rectus abdominis muscle from the posterior layer of the rectus sheath and observing the downward movement of the peritoneum (Figure 3). The same procedure was applied to the opposite side of the probe at the same position.

The total volume of local anesthetic solution was 50 mL, containing 0.25% bupivacaine, fentanyl 2 μg/mL, and dexamethasone 10 mg. Fentanyl was used for the rapid onset of block, and dexamethasone was used for the prolonged duration of analgesia. 10 mL of solution was injected at each block site—4 block sites for PIFB (2 in the right and 2 in the left parasternal areas). For RSB, 10 mL was used. 5 mL was injected on each side of the probe. Pain relief was achieved within 5 min of completing the block. Before the block, VAS was 10. After the block, it was 0. Before the block, the patient’s own tidal volume on expiration was 110–130 mL. After the block, it was around 210–230 mL. The weaning trial resumed the next day. All necessary measures were taken for a successful weaning trial. The patient was extubated after 48 h of block with stable hemodynamics. No more opioid was required. Only paracetamol was used as a rescue analgesic. Three days later, the patient was transferred from the ICU to the ward. Subsequently, the patient was discharged from the ward 1 week later. Postblock follow‐up was ensured. There was no hematoma, pain, or scar in the block site. Table 1 describes the timeline of clinical course and events.

**TABLE 1:** Timeline of clinical course and events.

Clinical timeline
Day 1: Initial extubation
Day 2: Reintubation
Day 2–15: Repeated weaning failures
Day 15: Transferred to Evercare ICU
Day 16: Weaning attempt failed due to severe pain
Day 17: Combined PIFB + RSB performed
Day 18–19: Weaning improved
Day 19: Extubated
Day 22: Transferred to the ward

## 3. Discussion

There are several regional blocks for the management of sternotomy pain. For this patient, reintubation and repeated weaning failure were due to severe pain, which restricted chest movement, impaired ventilation, and subsequently led to pneumonia and high opioid consumption. Combined bilateral PIFB and RSB provided complete pain relief. After the abolition of pain, discontinuation from mechanical ventilation was possible. Weaning from mechanical ventilation is a multifactorial‐dependent factor [5]. As this is a single case report, we do not know which factor contributed most, but the application of regional anesthesia definitely played a crucial role.

The PIFB provides reliable analgesia after midline sternotomy [7]. De La Torre is the one who described it first for breast surgery analgesia [8]. Several blocks can be used for median sternotomy pain management. The target is to block the anterior cutaneous branch of the thoracic 2 to 6 intercostal nerves. Several blocks have been proposed. These are the Erector Spinae Block (ESB), pectoral nerve block (PECS 1 & PECS 2), transverse thoracic muscle plane (TTP) block, and PIFB [9]. The ESB shows variable effect in blocking the anterior chest wall. The PECS block only provides lateral chest wall analgesia. Both the PIFB and TTP block provide similar efficacy for anterior chest wall analgesia. However, PIFB requires multilevel injections to cover a broader dermatomal distribution (T2−T6) for comprehensive poststernotomy pain coverage, as a single‐level injection may result in patchy analgesia due to inadequate dermatomal coverage [7]. Nevertheless, the safety margin is higher in the PIFB than in the TTP block, thereby reducing the risk of complications such as pneumothorax, lung injury, injury to the internal mammary vessels, and hematoma. Various studies have shown prominent results for PIFB [7, 9, 10].

Multiple chest drainage tubes are placed in the epigastric area through the rectus abdominis muscle, which also causes severe pain and distress for the patient, not adequately covered by the PIFB alone. Persistent epigastric pain may restrict abdominal wall movement and impair adequate ventilation. Bilateral RSB targets the anterior cutaneous branches of the lower thoracic nerves (T7−T12) and provides analgesia to the upper abdominal drain insertion sites [6, 10]. When bilateral RSB combines with PIFB, it not only provides comprehensive analgesia but also improves respiratory function [11]. In this case, although epigastric drains had been removed, the patient continued to experience epigastric tenderness and diaphragmatic splinting. A volume of 5 mL was chosen per side (10 mL total) for subxiphoid RSB, which was adequately spread within a confined fascial plane, visually confirmed by ultrasound, and proved effective for this patient. The addition of RSB complemented PIFB by eliminating both poststernotomy pain and epigastric tenderness, improving respiratory mechanics [12], and facilitating successful weaning from mechanical ventilation.

Opioids have been used as a primary tool for analgesia in cardiac surgical patients for a long time. Some patients require a higher dose of opioids, and it comes with lots of side effects such as respiratory depression, sedation, nausea, vomiting, and many others [1]. Regional anesthesia decreases dependency on opioids. As the world moves forward to reduce opioid dependence, the enforcement of the regional nerve block technique is going to help us reach that goal.

Neuraxial techniques such as thoracic epidural are particularly debatable in postcardiac surgical patients because of the threat of epidural hematoma, coagulopathy, and secondary neurological complications due to widespread usage of antiplatelet and anticoagulant drugs [6]. From this perspective, regional anesthesia is a relatively safer approach for pain management.

If poststernotomy pain is consistently present, it may cause severe respiratory comorbidity, with the obvious consequence of significantly affecting successful attempts to wean off mechanical ventilation, even after the optimal treatment of all parameters [2, 5]. Application of regional anesthesia not only reduces pain but, more importantly, enhances the breathing efforts, reducing median sternotomy‐related postoperative pulmonary morbidity.

In this case, the primary barrier to spontaneous breathing was limited chest expansion due to severe pain. Combined PIFB and RSB provided complete pain relief, improved respiratory effort, and facilitated weaning from mechanical ventilation, resulting in extubation.

Liberation from mechanical ventilation for a cardiac critical patient in whom weaning has failed repeatedly, following midline sternotomy, required many factors to be optimized. Despite optimum management of all key factors, the presence of severe poststernotomy pain led to respiratory complications, ultimately causing weaning failure from the mechanical ventilator. Implementation of regional anesthesia is a game‐changer here and reduces opioid requirements and opioid‐related complications. It is unknown which management factor contributed most to the weaning success. However, many factors and considerations should be taken into account for successful weaning from mechanical ventilation. For postcardiac surgical patients, excessive poststernotomy pain is one of them and can be successfully treated by implementing regional anesthesia.

## Author Contributions

Mohammad Zishan Uddin is responsible for the performance of the procedure, data collection, and the preparation of the initial and final manuscripts.

Maliha Karim is responsible for data collection and preparing the initial and final manuscripts.

Md. Abdul Karim, Niaz Ahmed, and Saiful Islam Khan are responsible for preparing the initial and final manuscripts.

## Funding

No funding was received for this work.

## Consent

Consent was taken for the usage of the image, clinical information, and publications.

## Conflicts of Interest

The authors declare no conflicts of interest.

## Patient Perspective

The patient experienced immediate pain relief, enabling her to undergo successful extubation.

## Data Availability

Data are available on request due to privacy/ethical restrictions.

## References

[1] Brennan T. J. , Pathophysiology of Postoperative Pain, Pain. (March 2011) 152, no. 3, S33–S40, 10.1016/j.pain.2010.11.005, 2-s2.0-79951576473. 21232860 PMC3073562

[2] Mazzeffi M. and Khelemsky Y. , Poststernotomy Pain: A Clinical Review, Journal of Cardiothoracic and Vascular Anesthesia. (December 2011) 25, no. 6, 1163–1178, 10.1053/j.jvca.2011.08.001, 2-s2.0-82255179409. 21955825

[3] Baumgarten M. C. , Garcia G. K. , Frantzeski M. H. et al., Pain and Pulmonary Function in Patients Submitted to Heart Surgery Via Sternotomy, Brazilian Journal of Cardiovascular Surgery. (2009) 24, no. 4, 497–505, 10.1590/s0102-76382009000500011. 20305923

[4] Kleiman A. M. , Sanders D. T. , Nemergut E. C. , and Huffmyer J. L. , Chronic Poststernotomy Pain: Incidence, Risk Factors, Treatment, Prevention, and the Anesthesiologist’s Role, Regional Anesthesia and Pain Medicine. (November 2017) 42, no. 6, 698–708, 10.1097/aap.0000000000000663, 2-s2.0-85032820130. 28937533

[5] Boles J. M. , Bion J. , Connors A. et al., Weaning From Mechanical Ventilation, European Respiratory Journal. (April 2007) 29, no. 5, 1033–1056, 10.1183/09031936.00010206, 2-s2.0-34248173856. 17470624

[6] Yamamoto T. , Seino Y. , Matsuda K. et al., Preoperative Implementation of Transverse Thoracic Muscle Plane Block and Rectus Sheath Block Combination for Pediatric Cardiac Surgery, Journal of Cardiothoracic and Vascular Anesthesia. (December 2020) 34, no. 12, 3367–3372, 10.1053/j.jvca.2020.07.041. 32800620

[7] Kumar A. K. , Chauhan S. , Bhoi D. , and Kaushal B. , Pectointercostal Fascial Block (PIFB) as a Novel Technique for Postoperative Pain Management in Patients Undergoing Cardiac Surgery, Journal of Cardiothoracic and Vascular Anesthesia. (January 2021) 35, no. 1, 116–122, 10.1053/j.jvca.2020.07.074. 32859487

[8] de la Torre P. A. , García P. D. , Álvarez S. L. , Miguel F. J. , and Pérez M. F. , A Novel Ultrasound-Guided Block: A Promising Alternative for Breast Analgesia, Aesthetic Surgery Journal. (January 2014) 34, no. 1, 198–200, 10.1177/1090820x13515902, 2-s2.0-84891866107. 24396082

[9] Liu H. , Emelife P. I. , Prabhakar A. et al., Regional Anesthesia Considerations for Cardiac Surgery, Best Practice & Research Clinical Anaesthesiology. (December 2019) 33, no. 4, 387–406, 10.1016/j.bpa.2019.07.008, 2-s2.0-85070517628. 31791558

[10] Wang L. , Jiang L. , Jiang B. et al., Effects of Pecto-Intercostal Fascial Block Combined With Rectus Sheath Block for Postoperative Pain Management After Cardiac Surgery: A Randomized Controlled Trial, BMC Anesthesiology. (March 2023) 23, no. 1, 10.1186/s12871-023-02044-w. PMC10035143 36959543

[11] Strumia A. , Pascarella G. , Sarubbi D. et al., Rectus Sheath Block Added to Parasternal Block May Improve Postoperative Pain Control and Respiratory Performance After Cardiac Surgery: A Superiority Single-Blinded Randomized Controlled Clinical Trial, Regional Anesthesia and Pain Medicine. (September 2025) 50, no. 9, 712–718, 10.1136/rapm-2024-105430. 38876800

[12] Huang Y. , Ouyang C. , He F. et al., Bilateral Parasternal and Rectus Sheath Blocks Reduce Pain Post-Cardiac Surgery: A Pilot Trial, Frontiers in Surgery. (February 2025) 12, 10.3389/fsurg.2025.1526890. PMC11882555 40052097

